# Applying quantitative spatial phenotypes analysis to the investigation of peltate glandular trichomes development pattern in *Perilla frutescens*

**DOI:** 10.1186/s13007-023-01072-4

**Published:** 2023-08-25

**Authors:** Zheng Jiang, Peina Zhou, Yongfang Shao, Qianqian Zhang, Wei Yue, Cheng Qu, Qinan Wu

**Affiliations:** 1 College of Pharmacy, Nanjing University of Chinese Medicine, Nanjing, 210023 China; 2 Collaborative Innovation Center of Chinese Medicinal Resources Industrialization, Nanjing, 210023 China; 3 National and Local Collaborative Engineering Center of Chinese Medicinal Resources Industrialization and Formulae Innovative Medicine, Nanjing, 210023 China

**Keywords:** Growth axis, Linear regression equation, Leaf veins, Peltate glandular trichomes, *Perilla frutescens*, Spatial phenotype

## Abstract

**Background:**

Glandular trichomes, often referred to as “phytochemical factories”, plays a crucial role in plant growth and metabolism. As the site for secretion and storage, the development of glandular trichomes is related to the dynamic biosynthesis of specialised metabolites. The study aims to explore the relationship between spatial phenotype and dynamic metabolism of glandular trichomes, and establish a novel approach for the exploration and study of the regulatory mechanism governing the development of glandular trichomes.

**Results:**

In this study, we proposed a technical route based on the relative deviation value to distinguish the peltate glandular trichomes (PGTs) from the background tissues and extract their spatial phenotype. By defining glandular trichome developmental stages based on the leaf vein growth axis, we found that young PGTs were densely distributed near the proximal end of growth axis of the leaf veins, where perillaketone, a primary metabolite of PGTs, is predominantly accumulated. Conversely, mature PGTs are typically found near the distal end of the mid-vein growth axis and the lateral end of the secondary vein growth axis, where the accumulation rate of isoegomaketone and egomaketone exceeds that of perillaketone in PGTs. We further identified spatial phenotypic parameters, L_sum_ and d, as independent variables to construct a linear regression model that illustrates the relationship between the spatial phenotypes and metabolite content of PGTs, including perillaketone (R^2^ = 0.698), egomaketone (R^2^ = 0.593), isoegomaketone (R^2^ = 0.662) and the sum of the amount (R^2^ = 0.773).

**Conclusions:**

This model proved that the development of PGTs was correlated with the growth of the entire leaf, and the development stage of PGTs can be identifined by spatial phenotypes based on the leaf veins. In conclusion, the findings of this study enhance our understanding of correlation between spatial phenotype and development of glandular trichomes and offer a new approach to explore and study the regulatory mechanism of glandular trichome development.

**Supplementary Information:**

The online version contains supplementary material available at 10.1186/s13007-023-01072-4.

## Background

Trichomes, epidermal extensions found on epidermal cells, are ubiquitous in the majority of plant species. Various morphologies and functions of trichomes have been identified across different plant species. Among various trichomes, glandular trichomes (GTs), known for their secretory functions, are commonly termed “phytochemical factories” and play a critical role in plant defence against external stress [1]. The biochemical metabolism of various metabolites, including terpenoids, simple phenols, flavonoids, glycerides, and alkaloids, occurs in GTs [2, 3]. These specialised metabolites have broad commercial applications, spanning from pesticides and perfumes to food additives and pharmaceuticals [4–8]. As the primary site for secretion and storage, the growth and development of GTs are related to the dynamic biosynthesis of specialised metabolites [9]. Therefore, studying the dynamic metabolism of development-associated metabolites during GT development could significantly deepen our understanding of the regulatory networks involved.

The regulation of GT development is a complex process influenced by multiple factors such as genotype [10], abiotic and biotic stresses [11, 12], and regulatory networks operating within and between different trichome types [13]. Additionally, different cellular and molecular processes occurring at various developmental stages, influenced by spatiotemporal differential gene expression, play a crucial role in determining the GTs’ life cycle [9, 14–17].

Consequently, acquiring GTs in which the developmental stage can be identified is a key technical requirement for investigating GT development. Throughout the GT development, a series of morphological transformations occur, including variations in cell dimensions, proliferation of secretory cells, and progressive release of secondary metabolites [15, 18, 19]. Morphological observations provide an intuitive basis for determining GT developmental stages, a method widely adopted by researchers [1, 9, 20–22]. Despite its wide applicability, this approach has limitations due to the lack of synchrony in GT development within the same organ or plant and insufficient precision in the time interval between developmental stages. Defining trichome development stages according to the developmental stages of the organs or plant individuals to which they are attached was also a common strategy in previous studies. This approach has great operability and wide applicability, and has significantly contributed to our understanding of GTs development [7, 20]. However, such an approach also has inherent limitations. On the one hand, GT development in the same organ or plant individual is not completely synchronous [21–23]. Therefore, research on organs or individual plants at specific growth stages indicates only the average metabolism of GTs, leading to a lack of representativeness. On the other hand, the time interval between the developmental stages of organs and plant individuals may not be sufficiently precise, resulting in the exclusion of dynamic metabolic details of GTs during shorter periods of growth. Additionally, in some species, GTs attached to mature leaves exhibit consistent developmental stages, possibly due to the brief duration of the various developmental stages of GTs before maturation [23, 24]. Therefore, the definition of the GT development stages on a more detailed spatial scale will be beneficial for our comprehension of GT development.

With the advancement in research on cell interaction, microenvironment, and metabolism, the spatial phenotypes of specific cells have gained increased attention [25–27]. Spatial multi-omics techniques have facilitated investigations at the cellular level, expanding the research object’s spatial scale [25, 28, 29]. Evidence from molecular biology, morphometry and metabolomics obtained by utilizing spatial multi-omics techniques has demonstrated that cells with the similar spatial distribution typically exhibit similar developmental stages [30]. According to the growth direction of tissues or organs, developmental trajectories can be established among different zones where cells are located [28]. Consequently, the developmental trajectories and developmental gradient of GTs can also be explored, according to the zones where GTs are attached and the extension direction of leaves.

The extension direction and shape formation of leaves can be explained perfectly by tissue-wide polarity field theory [31]. Studies on the polarity field of *Arabidopsis* leaf epidermal cells have revealed two main polarity field directions during leaf growth and shape formation: one from the leaf-base to leaf-top (proximate-distal axis) and the other from leaf-middle to the leaf-margin at a certain angle to the proximate-distal axis (median-lateral axis) [32, 33]. In *dicotyledons*, these axes are almost consistent with the growth direction of the mid-veins and secondary veins, as shown in Additional file 1 [34]. This demonstrates the potential of leaf veins to serve as a reference frame for identifying the developmental gradient of each leaf zone across different leaf zones, thereby providing a means to mark the developmental stages of GTs.

*Perilla frutescens* (L.) a significant cooking spice and medicinal herb, is prevalent in various Asian countries, including China, Thailand, India, and Japan. [35]. Contemporary research has shown that *P. frutescens* essential oil exhibits a broad range of biological activities including antibacterial, antioxidant, antidepressant, anti-inflammatory and intestinal-promoting [36–39]. Prior studies have identified the presence of three types of GTs on the leaves and stems of *P. frutescens*, with peltate glandular trichomes (PGTs) being the most abundant and serving as key storage structures for essential oils. [40, 41]. Consequently, given their flat surfaces and lack of shape disparity, *P. frutescens* leaves provide ideal models for investigating the spatial phenotype of GTs.

Accordingly, in this study, we propose a technical approach that leverages the relative deviation value to distinguish PGTs from the surrounding tissues and extract their spatial phenotype. Utilizing the growth axis of leaf veins as a reference frame, we were able to define distinct zones of PGTs and quantitatively characterized their spatial phenotypes. Subsequently, a linear regression model that could explain the development pattern of PGTs was established with spatial quantitative phenotypic parameters as the independent variables. Through the joint analysis of PGT spatial phenotypes and developmental gradients, we found that the development of PGTs was correlated with the growth of the entire leaf and the developmental stages of PGTs can be identified by their spatial phenotypes based on the leaf veins. The striking variation of the volatile oil metabolism in *P. frutescens* PGTs was also found during the maturation. These findings enhance our understanding of correlation between spatial phenotype and development of GTs and provide an approach for the exploring and studying of the regulatory mechanism governing the development of glandular trichomes.

## Materials and methods

### Plant materials

*Perilla frutescens* (L.) accessions seeds were acquired from the Anguo City medicine market in Hebei Province, China, and kept at 4 ℃ in our laboratory. Using plant nutritive soil (Miracle-Gro nutritive soil, Scott’s Miracle-Gro, Marysville, OH, USA), the seedlings were planted in a phytotron in the mid-May 2022 under carefully controlled conditions (day/night, 10/14 h, 30/25 ℃, 60% RH) in the greenhouse. Following a two-month cultivation period, when the plant heights reached approximately 50 cm, the first and second nodes of the emerged leaves (from shoot to root) were collected for image acquisition and Gas chromatography Mass Spectrometry (GC–MS) analysis.

### Stereomicroscope analysis of trichomes in *P. frutescens*

The morphology and distribution of trichomes on ten leaves collected from ten individual plants were evaluated using a stereomicroscope (SetREO Discovery, V20, Zeiss, Jena, Germany). For each leaf, ten observation fields were randomly selected on both the adaxial and abaxial surfaces, with a size of 1 × 1 mm, using a stereomicroscope. The dimensions of all types of GTs in each observation field were measured. The dimensions of GT head cells were measured using ZEN Microscopy Software (Zeiss, Jena, Germany).

### Image acquisition and processing of PGTs in *P. frutescen*s

**Fig. 1:**
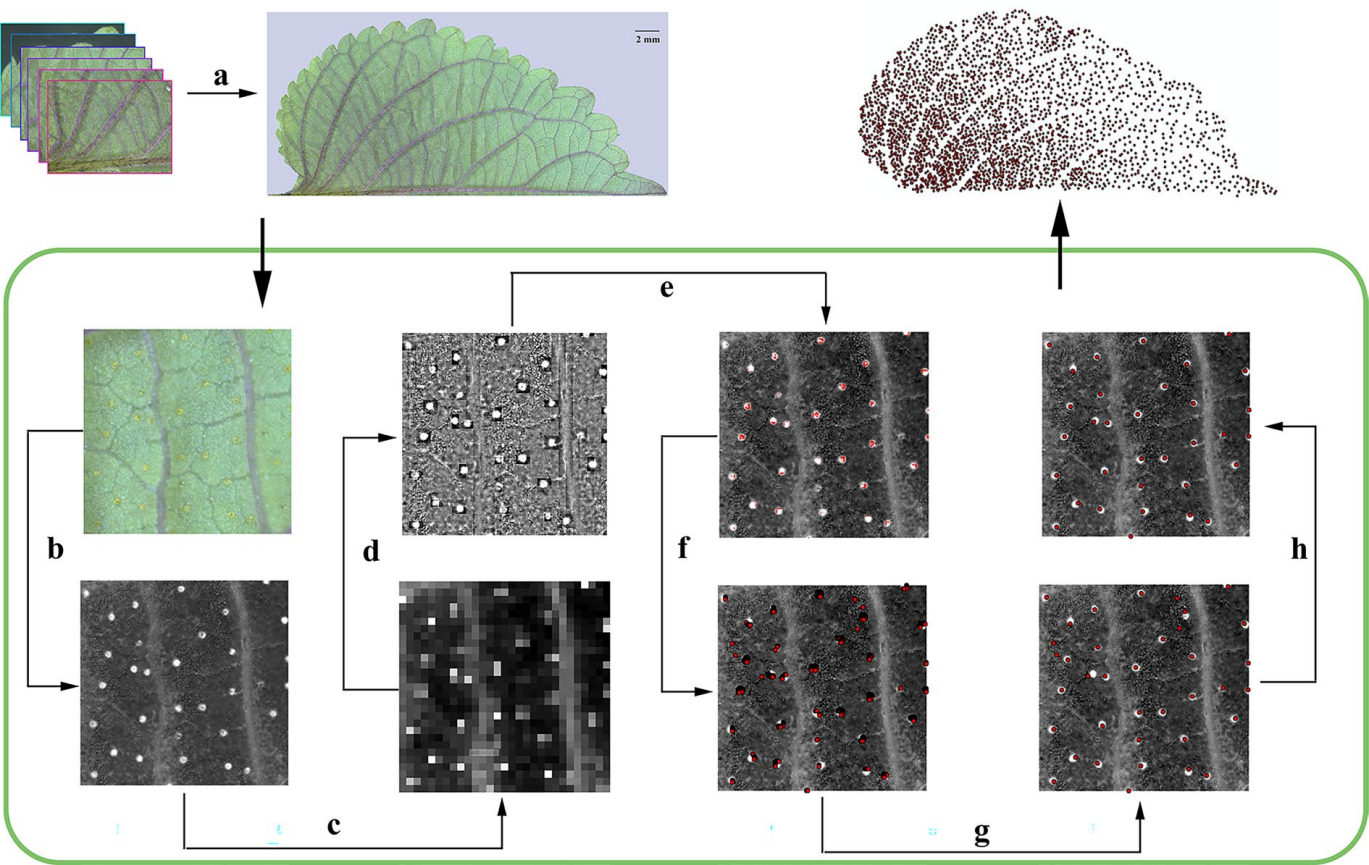
Technical route of point vector map generation of *P. frutescens* PGTs distribution*.* **a** Photomerge; **b** Adding a black and white mask; **c** Generating a map of the mean pixel values (16 × 16 pixels) map; **d** Calculating and generating a vector graph of RD values; **e** Setting the threshold to obtain a PGTs distribution raster map; **f** Converting the PGTs distribution raster map to the point vector map; **g** Aggregating points; **h** Artificial modification

The leaves of *P. frutescens* exhibit an axisymmetric arrangement along the mid-vein. Therefore, the microscopic features of PGTs on one side of the mid-vein were investigated. Leaf images were captured using a stereomicroscope (SetREO Discovery, Zeiss) and subsequently processed into a merged leaf image using Adobe Photoshop (version 13.0, Adobe Systems Incorporated, San Jose, CA, USA) **(**Fig. 1a**)**.

To convert the merged image into a black-and-white image, we utilized the black-and-white mask function of Adobe Photoshop (version 13.0, Adobe Systems Incorporated). The red and yellow channel setting parameters were set to 300, while the blue, cyan, and green channel parameters were set to − 200 **(**Fig. 1b**)**. Subsequently, the relative deviation (RD) of each pixel value in each unit was calculated according to Eq. (1) by Arcmap (version 10.8, Environmental Systems Research Institute, Redlands, CA, USA) with a pixel number of 16 × 16 processing units, resulting in a raster image of the RD **(**Fig. 1c, d). The RD threshold was determined through direct observation and pixels exceeding the RD threshold were designated as null, resulting in a raster image of the PGT distribution **(**Fig. 1e**)**. Subsequently, this raster image was converted into a point vector map (Fig. 1f). Vector points separated by a distance of eight pixels were aggregated into a single point (Fig. 1g). Lastly, the point vector map was manually modified to align with the actual PGTs distribution **(**Fig. 1h**)**.

1 $$Relative \,Deviation \left(\%\right)=\frac{V-{V}_{m}}{{V}_{m}}\times 100\%.$$

In the formula, V is the value of each pixel, and V_m_ represents the average value of 256 pixel values in the range of 16 × 16 pixels.

### The spatial phenotypes of PGTs in* P. frutescens*

**Fig. 2:**
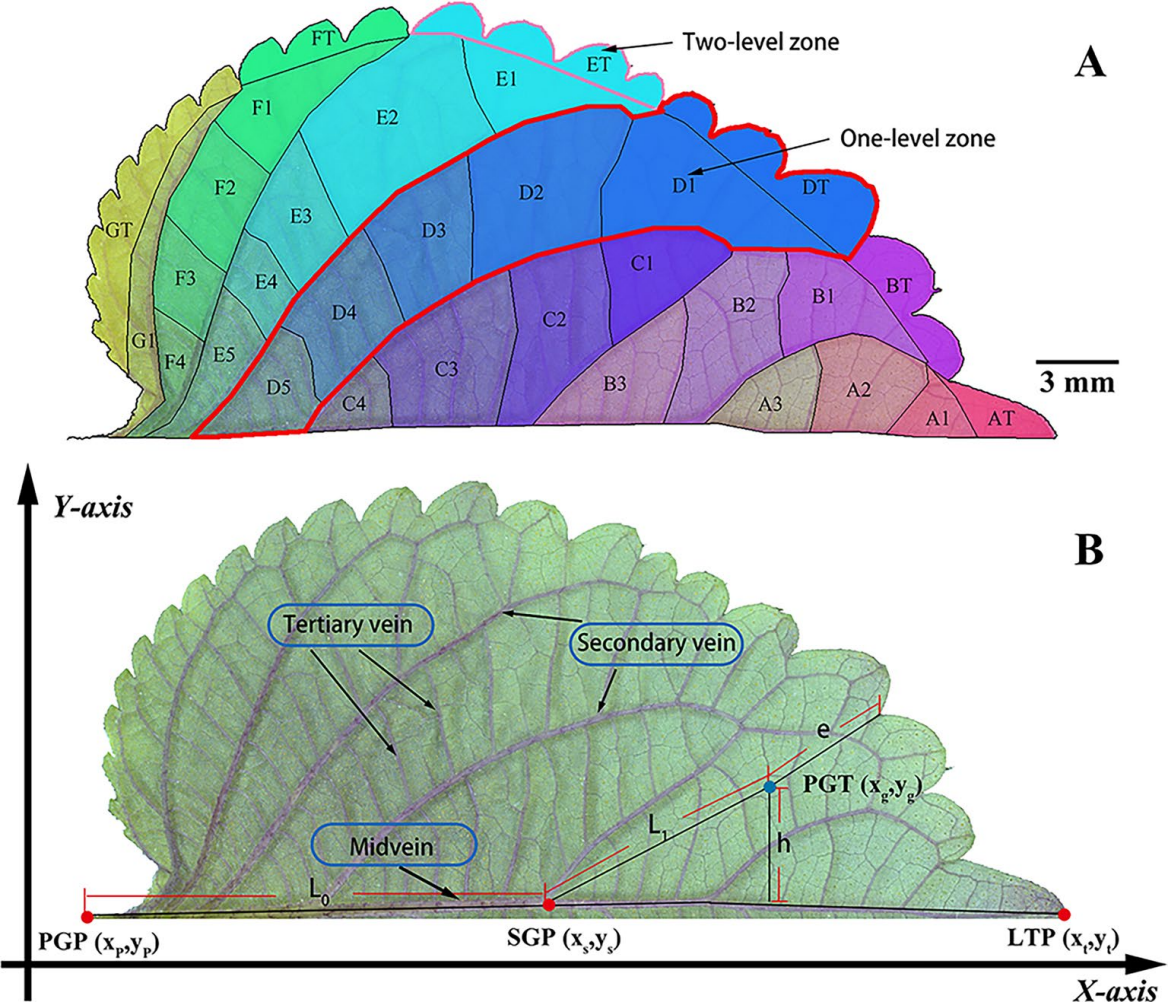
Spatial phenotypic parameters of PGTs. **A** The zone of the PGTs; **B** definition of spatial phenotypic parameters

The kernel density package in Arcmap (version 10.8, Environmental Systems Research Institute) was utilized to analyse the distribution density of PGTs. Spatial phenotypes of each PGT were defined according to the coordinates generated from the image using Arcmap (version 10.8, Environmental Systems Research Institute). The coordinates of PGT, primary growth point (PGP), secondary growth point (SGP), and leaf top point (LTP) were represented as (x_g_, y_g_), (x_p_, y_p_), (x_s_, y_s_), and (x_t_, y_t_), respectively (Fig. 2). Spatial phenotypic parameters were computed using the formula outlined in Table 1.

**Table 1:** The calculated formula of spatial phenotypic parameters

No	Name	Abbreviation	Calculated formula
1	Primary growth distance	L_0_	${\mathrm{L}}_{0}=\sqrt{{({x}_{s}-{x}_{p})}^{2}+{({y}_{s}-{y}_{p})}^{2}}\times \rho$
2	Secondary growth distance	L_1_	${\mathrm{L}}_{1}=\sqrt{{({x}_{g}-{x}_{s})}^{2}+{({y}_{g}-{y}_{s})}^{2}}\times \rho$
3	Sum of growth distance	L_sum_	${L}_{Sum}={L}_{0}+{L}_{1}$
4	Growth angle	θ	$\theta = {\text{arctan}}\left( {\left| {\frac{{\frac{{(y_{g} - y_{s} )}}{{(x_{g} - x_{s} )}} - \frac{{(y_{t} - y_{s} )}}{{(x_{t} - x_{s} )}}}}{{1 - \frac{{(y_{g} - y_{s} )}}{{(x_{g} - x_{s} )}}*\frac{{(y_{t} - y_{s} )}}{{(x_{t} - x_{s} )}}}}} \right|} \right) \times \frac{\pi }{2} \times 90^{^\circ }$
5	Vertical growth distance	h	$h=\mathrm{cos}(\uptheta )\times {L}_{1}$
6	Edge distance	e	/
7	Nearest distance	d	/

$\uprho$ is the actual distance represented by the unit coordinate value, which was 0.538414 mm; L_0_ is the primary growth distance, which is the distance between the PGT and SGP; L_1_ is the secondary growth distance, which is the distance between SGP and PGT; θ is the growth angle, which is the angle between the line from SGP to PGT and the mid-vein; h is the vertical growth distance, which is the vertical distance between PGT and the mid-vein; e is the edge distance, which is the nearest distance between PGT and the leaf margin; d is the adjacent distance, which is the average distance between the target PGT and its three adjacent PGTs; L_sum_ is the comprehensive growth distance, which is the sum of primary growth distance and secondary growth distance (Fig. 2)

The “Average Nearest Neighbour” analysis package was employed to calculate edge distance (e) and adjacent distance (d). The resultant density heatmap of *P. frutescens* PGT distribution was obtained by the ‘Kernel Density’ package of Arcmap (version 10.8, Environmental Systems Research Institute).

### The zoning scheme of PGTs in *P. frutescens*

The *P. frutescens* leaf was divided along the mid-vein into one-level zones, which were sequentially labeled as A, B, C, D, and so on, from the top of the leaf to the base. (Fig. 2A). Each one-level zone was further subdivided into two to five zones along the secondary veins, creating two-level zones labeled as A1, A2, and A3, based on their distance from the mid-vein (Fig. 2A). Zones closer to the leaves margin were labeled as AT, BT, DT, ET, FT, and GT. (Fig. 2A). The average value of spatial phenotypes of PGTs located in the same zone were calculated using Arcmap (version 10.8, Environmental Systems Research Institute).

### Sample preparation, chemicals, chemicals and GC–MS conditions

The internal standard solution was obtained by dissolving 100 μL of n-hexadecane in 50 mL of ethyl acetate, and 1 mL of this solution was dissolved in 100 mL of ethyl acetate. The standards (perillaketone, isoegomaketone, and β-caryophyllene) were dissolved in this internal standard solution to obtain concentrations of 17.8 mg/mL, 20.8 mg/mL, and 18.0 mg/mL, respectively. Consequently, a standard curve solution was obtained by gradient dilution of the aforementioned standards solution.

The leaves were divided into different parts according to the zoning scheme using iridectomy lancet and microsurgical tweezers. To prevent the errors in volatile component determination caused by damage to the PGTs due to the division, the PGTs on each individual part were observed and counted under a stereomicroscope (SetREO Discovery, Zeiss). The number of PGTs obtained was used to calculate the compound content in a single PGT. A total of 240 divided zones of ten leaves from ten plant individuals were measured. Each zone of the *P. frutescens* leaf was homogenized with 200 μL of the internal standard solution and five stainless steel beads (diameter 3 mm) using a JXFSTPRP-24 homogenizer (Shanghai Jingxin Industrial Development Co., Ltd., Shanghai, China) at 60 Hz for 30 s. The resulting mixture was centrifuged at 13,000 rpm for 10 min, and the supernatant was filtered using 0.22 μm microporous filter membranes prior to the GC–MS analysis.

Perillaketone and isoegomaketone standards were purchased from Chengdu DeSiTe (Chengdu DeSiTe Biological Technology Co., Ltd, Chengdu, China), while the β-caryophyllene standard was purchased from Aladdin (Shanghai Aladdin Biochemical Technology Co., Ltd, Shanghai, China).

An Agilent 7893A gas chromatograph coupled with an Agilent 7000C mass spectrometer (Agilent Technologies, Santa Clara, CA, USA) in electron impact mode was used to identify volatile oil compounds. The mass spectra were scanned from 30 to 500 amu. Agilent DB-5 ms (30 m × 250 μm × 0.25 μm; Agilent Technologies, Santa Clara, CA, USA) was used to separate the volatiles. The injector, gasification chamber, and detector temperatures were set at 200 ℃, 250 ℃ and 250 ℃, respectively. The column temperature was initially 50 ℃ (held for 3 min), then raised to 125 ℃ at a rate of 10 ℃/min, and finally raised to 190 ℃ at 15 ℃/min (held for 1 min). Helium was used as the carrier gas at a flow rate of 2.0 mL/min with an injection volume of 1.5 μL without splitting.

### Data analysis

#### Identification and quantification of compounds

The identification of perillone, isoegomaketone, egomaketone, and β-caryophyllene were conducted by comparing their retention times and recorded mass spectra with those of standard substances. Other components were identified by matching their recorded mass spectra with the National Institute of Standards and Technology mass spectral library (NIST 14.0L). The contents of perillone, isoegomaketone, egomaketone, and β-caryophyllene in each zone were determined using the internal standard method. The egomaketone content was calculated using isoegomaketone as a standard substance. By combining the number of PGTs in each zone, the average contents of perillone, isoegomaketone, egomaketone, and β-caryophyllene in single PGTs were calculated and analysed. The total amounts of perillone, isoegomaketone, and egomaketone were calculated using Eq. (2).

2 $$\mathrm{Total \,amount}\,\left(\mathrm{nmol}\right)=\frac{{m}_{i}}{164.2}+\frac{{m}_{e}}{164.2}+\frac{{m}_{p}}{166.2}$$

In the formula, m_i_ is the weight of isoegomaketone (ng), m_e_ is the weight of egomaketone (ng), and m_p_ is the weight of perillone (ng).

#### Principal component analysis (PCA) and linear regression analysis

PCA of the spatial phenotypic parameters of each leaf zone was performed using SIMCA-P (vension.14.1, Umetrics AB, Umea, Sweden). Single and multiple linear regression models were constructed to describe the relationship between each spatial phenotypic parameter and the volatile content of an individual PGT using SPSS (vension.26.0, IBM SPSS Statistics, Armonk, NY, USA).

## Results

### Morphology and distribution of trichomes in *P. frutescens*

**Fig. 3:**
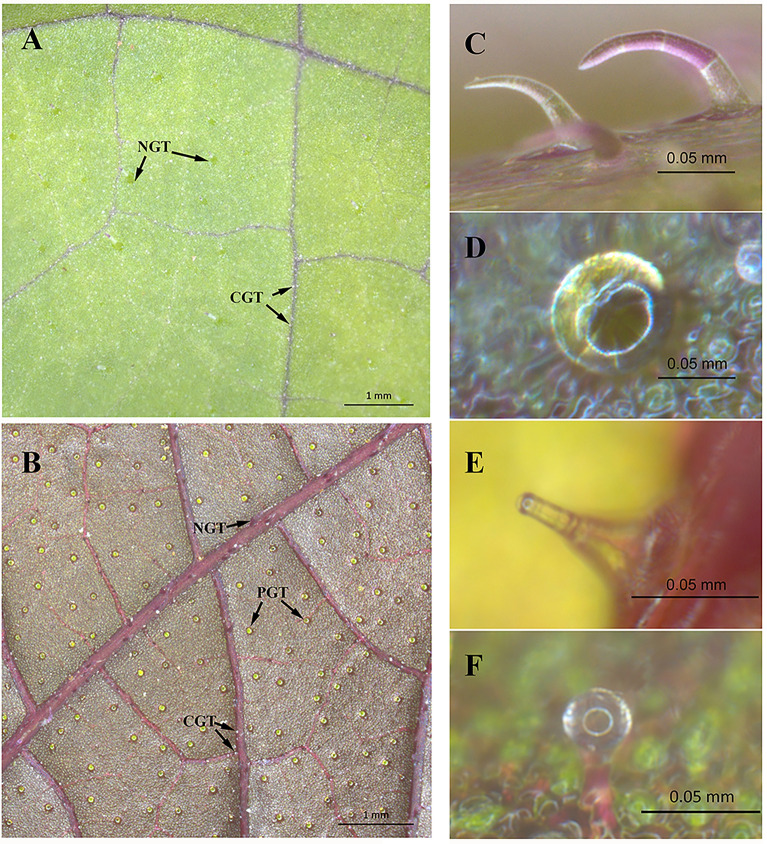
The morphology of glandular trichomes in *P. frutescens.* **A** adaxial surface of *P. frutescens* leaves; **B** abaxial surface of *P. frutescens* leaves; **C** non-glandular trichome (NGT); **D** peltate glandular trichome (PGT); **E** digitiform glandular trichome (DGT); **F** Capitate glandular trichome (CGT)

In the leaves of *P. frutescens*, four types of trichomes were identified, three of which were considered to possess secretory functions: PGTs, capitate glandular trichomes (CGTs), and digitiform glandular trichomes (DGTs) (Fig. 3). These GTs in *P. frutescens* were structurally analogous to those present in other *Lamiaceae* plants, displaying a tripartite organization consisting of basal, stalk, and head cells [20, 42, 43]. In this study*,* we found *P. frutescens* PGTs are spheroid structures that accumulate oily substances during their maturation stages. They are distributed on the abaxial surface of the leaf, specifically on the epidermal cells surrounded by the veins, with a few distributed on the veins themselves. CGTs are either spherical or nearly spherical and are found on the veins of both surfaces of the *P. frutescens* leaf. DGTs exhibit a finger-like structure with head cells smaller than the stalk cells, and are sparsely distributed on the veins of the abaxial surface of the leaf.

There were significant variations in the size and quantity of different types of GTs in *P. frutescens*. PGTs stood out with the largest volume and highest quantity (Table 2, Additional file 2). The head cells which primary store volatile oils, can be approximated as spherical. The volume ratio of volatile oils present in different GTs can be determined through the application of Eq. (3). The volume ratio of volatile oils in PGTs to CGTs was 33.14, and to DGTs was 2829.6.

**Table 2:** The quantity and head cells dimensions of various GTs in* P. frutescens*

GT type	Quantity of GTs		Head cells diameter (μm)	
	Adaxial	Abaxial	Adaxial	Abaxial
PGTs	0	657	N/A	72.4 ± 6.0
CGTs	347	167	25.3 ± 3.5	24.5 ± 2.7
DGTs	0	43	N/A	12.7 ± 0.7

3 $$\frac{{V_{P} }}{{V_{C} }} = \frac{{\frac{4}{3}\pi R_{P}^{3}  \times Q_{P} }}{{\frac{4}{3}\pi R_{C}^{3}  \times Q_{C} }} = \left( {\frac{{R_{P} }}{{R_{C} }}} \right)^{3}  \times \frac{{Q_{P} }}{{Q_{C} }} = \left( {\frac{{D_{P} }}{{D_{C} }}} \right)^{3}  \times \frac{{Q_{P} }}{{Q_{C} }} $$

In the formula, V_P_ is the volume of PGT, V_C_ is the volume of CGT or DGT, R_P_ is the radius of PGT, R_c_ is the radius of CGT or DGT, Q_P_ is the quantity of PGT, Q_C_ is the quantity of CGT or DGT, D_P_ is the diameter of PGT, and D_c_ is the diameter of CGT or DGT.

### Identification of compounds in volatile oils

The volatile constituents of different zones were detected using GC–MS, and a total of 19 compounds were identified (Table 3). Among these compounds, perillaketone, isoegomaketone, egomaketone, and β-caryophyllene were found to be the primary constituents of *P. frutescens* volatile oils, accounting for 48.6%, 13.6%, 2.2%, and 3.8% of the identified compounds respectively. Cumulatively, these four compounds represented 68.3% of the identified compounds. As shown in Additional file 3, the compounds identified in the GC–MS analysis were well separated. Peaks 1 to 5 corresponded to perillaketone, isoegomaketone, egomaketone, β-caryophyllene, and n-hexadecane, respectively. Perillaketone, isoegomaketone, and egomaketone had the highest intensity of fragment ions at M/Z 95.1, while β-caryophyllene and n-hexadecane had the highest intensity of fragment ions at M/Z 132.8 and 71.1, respectively. Consequently, these fragment ions were selected as quantitative ions.

**Table 3:** The compounds identified in the volatile oils of *P. frutescens*

No	Retention time (min)	Name	Formula	Molecular mass (g/mol)	Relative content (%)	Identification mode
1	3.083	*o*-Xylene	C_8_H_10_	106.1	1.3%	NIST
2	3.278	Styrene	C_8_H_8_	104.1	1.1%	NIST
3	3.306	Ethylbenzene	C_8_H_10_	106.1	1.9%	NIST
4	4.22	1-octen-3-ol	C_8_H_16_O	128.1	0.6%	NIST
5	5.256	Dodecane, 2,6,10-trimethyl-	C_15_H_32_	212.3	0.9%	NIST
6	7.222	Dodecane	C_12_H_26_	170.2	0.7%	NIST
7	7.465	Benzaldehyde, 2,5-dimethyl-	C_9_H_10_O	134.1	0.6%	NIST
8	7.899	Perillaketone	C_10_H_14_O_2_	166.2	48.6%	RS/NIST
9	8.084	Tetradecane, 2,6,10-trimethyl-	C_17_H_36_	240.3	1.3%	NIST
10	8.287	Dodecane, 2,6,11-trimethyl-	C_15_H_32_	212.3	1.6%	NIST
11	8.396	Octadecane, 3-ethyl-5-(2-ethylbutyl)-	C_26_H_54_	366.4	0.5%	NIST
12	8.457	Egomaketone	C_10_H_12_O_2_	164.2	2.2%	RS/NIST
13	8.539	Isoegomaketone	C_10_H_12_O_2_	164.2	13.6%	RS/NIST
14	8.835	Heptacosane	C_27_H_56_	380.4	1.2%	NIST
15	9.639	Tetradecane	C_14_H_30_	198.2	2.2%	NIST
16	9.864	Heptadecane, 9-hexyl-	C_23_H_48_	324.4	0.7%	NIST
17	9.94	β-Caryophyllene	C_15_H_24_	204.2	3.8%	RS/NIST
18	10.575	Bicyclo[3.1.1]hept-2-ene, 2,6-dimethyl-6-(4-methyl-3-pentenyl)-	C_15_H_24_	204.2	11.0%	NIST
19	10.726	Phenol, 2,4-bis(1,1-dimethylethyl)-	C_14_H_22_O	206.2	6.0%	NIST

RS: Identified according to reference substance, NIST: Identified according to National Institute of Standards and Technology mass spectral library

### The spatial phenotype analysis of* P. frutescens* PGTs

Utilizing the technical route depicted in Fig. 1 to calculate the RD values, the dissimilarities between PGTs and background tissues were effectively discriminated, thereby enabling the extraction of position information and spatial phenotypes of the PGTs with high precision.

**Fig. 4:**
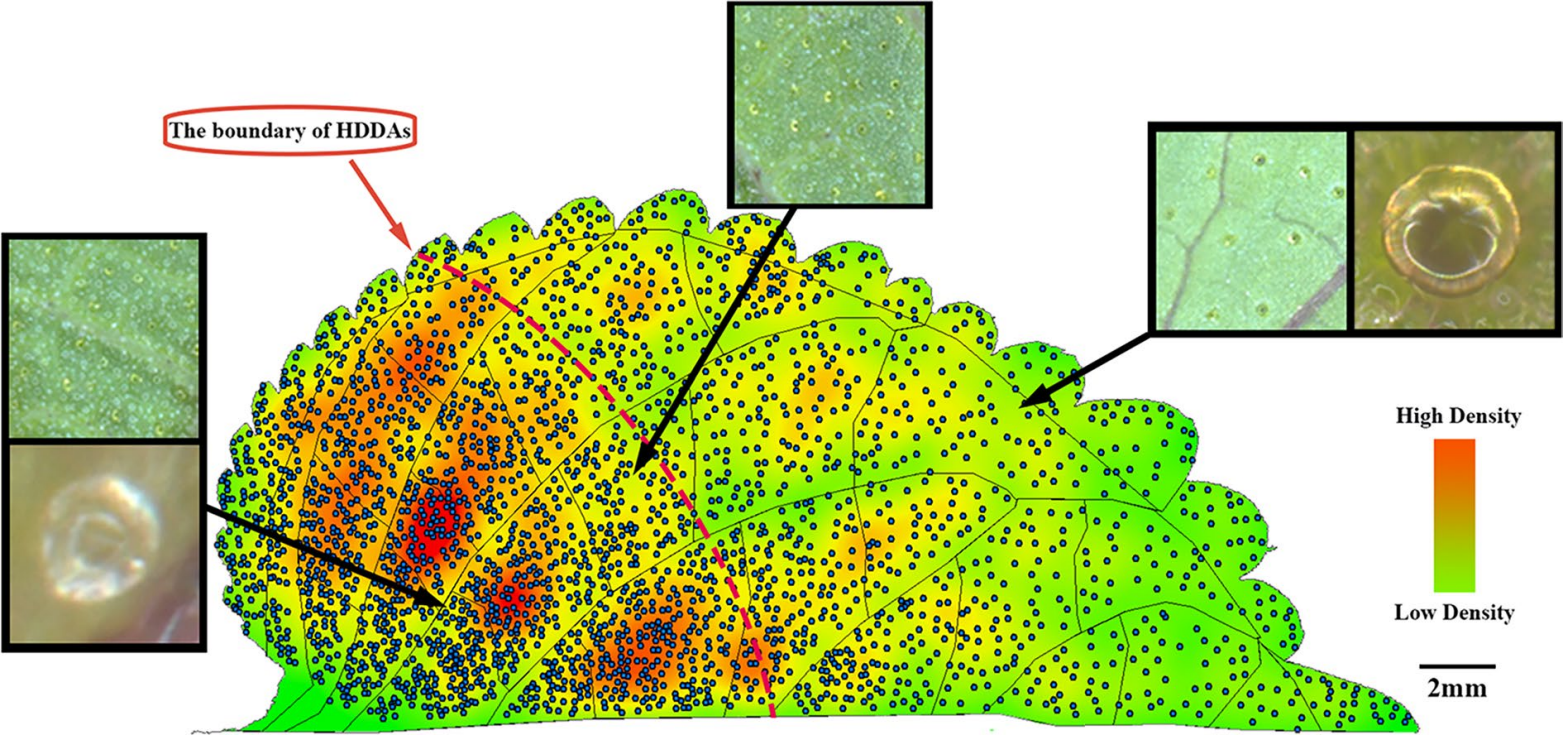
Density heat map of *P. frutescens* PGTs distribution

PGTs demonstrated a high-density distribution in certain zones, and the high-density distribution areas (HDDAs) demonstrated a certain distribution pattern across various zones. The HDDAs were larger in the first-level zone closer to the leaf base compared to those farther away. In the first-level zone nearest to the top of the leaf, PGTs were distributed almost uniformly without the discernible HDDAs (Fig. 4). Within the same first-level zone, the PGT density was higher in the secondary-level zone closer to the mid-vein, while the zone farther away from the mid-vein had a lower PGT density. Intriguingly, PGT phenotypes varied in areas with different distribution densities. Specifically, PGTs in low-density areas primarily displayed mature characteristics, such as large size and a significant amount of brown-yellow volatile oil. Conversely, PGTs in HDDAs were predominantly young and small, with transparent volatile oils. In medium-density areas, both mature and young PGTs were found to be interspersed. The boundary of HDDAs exhibited a curved line bending towards the proximal of the leaf.

### Compound distribution in different zones

**Fig. 5:**
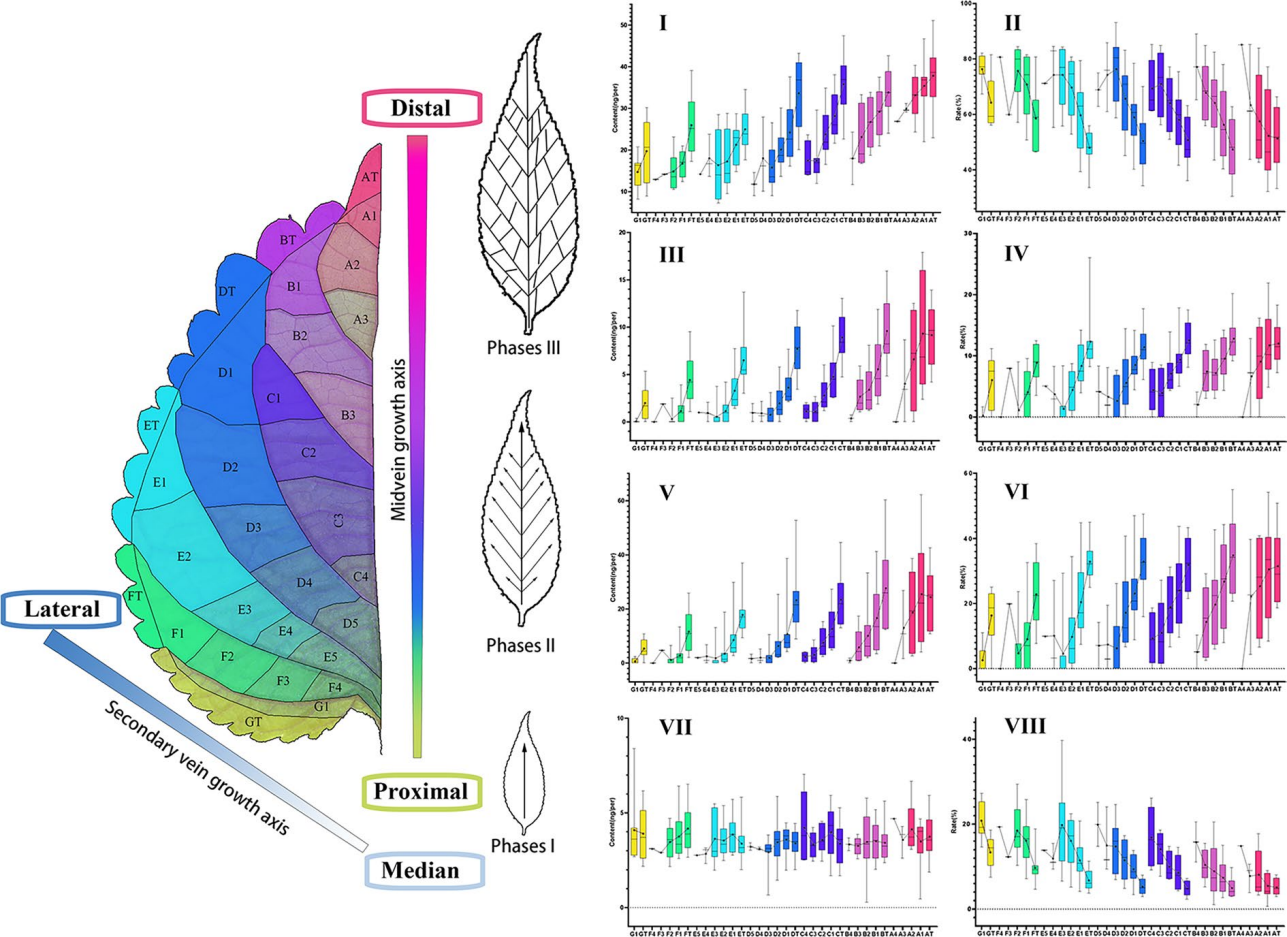
The contents variation of the main compounds in PGTs from different divisions. **I** The content of perillaketone in single PGT; **II** The proportion of perillaketone in volatile oils; **III** The content of egomaketone in single PGT; **IV** The proportion of egomaketone in volatile oils; **V** The content of isoegomaketone in single PGT; **VI** The proportion of isoegomaketone in volatile oils; **VII** The content of β-caryophyllene in single PGT; **VIII** The proportion of β-caryophyllene in volatile oils. The leaf shown in the Figure is sample 1

The contents of the four main compounds in the PGTs were quantitatively analysed to explore their dynamic patterns in different zones. The findings demonstrated that PGTs in the first-level zones towards the base of the leaf, such as the G and F zones, had lower contents of perillaketone, isoegomaketone, and egomaketone, while those zones near the top of the leaf, such as the A and B zones, had higher contents (Fig. 5). Furthermore, the contents of the three aforementioned compounds in the PGTs increased with distance from the mid-vein within the same first-level zone. Some PGTs near the mid-vein were found to lack isoegomaketone or egomaketone entirely. As the content increased, the proportion of isoegomaketone and egomaketone in the volatile oils gradually increased, while the proportion of perillaketone gradually decreased.

In contrast, the content of β-caryophyllene in individual PGT across different zones did not exhibit an obvious trend, and its proportion in volatile oils gradually decreased from zone G to A (Fig. 5).

### PCA of spatial phenotypic parameters

To explore the relationship among the spatial phenotypic parameters of L_0_, θ, e, L_1_, h, L_sum_, and d, PCA analysis was performed on all parameters (Additional file 4). The results showed that the abscissa axis was explained by L_sum_, d, and h (PC1, 52.2%), and the longitudinal axis was explained by e, L_0_, and θ (PC2, 23.7%). The details are presented in Table 4. The results also indicated an association between the abscissa axis and the concentrations of perillaketone, isoegomaketone, and egomaketone (Additional file 4: Fig. S3A–C). However, the variance in β-caryophyllene content could not be accounted for by the spatial phenotypes of PGTs, as it was not associated with PC1 or PC2 (Additional file 4: Fig. S3D).

**Table 4:** The PCA loading coefficient of the spatial phenotypes of PGTs

Component	Proportion of variance	loading coefficient						
		L_0_	θ	e	L_1_	h	L_sum_	d
PC1	52.2	0.2084	− 0.3368	− 0.1094	0.4061	0.4664	0.4983	0.4476
PC2	23.7	0.5186	− 0.4839	0.5342	− 0.4081	− 0.1904	0.0934	− 0.0104

The spatial phenotypic parameters of L_sum,_ d, and h were found to be useful in explaining the variance in the sum amount of perillaketone, isoegomaketone, and egomaketone due to the strong correlation between the total amounts of these three compounds and PC1 (Table 4, Additional file 5).

### Linear regression model analyses

The interrelationship among L_sum_, d, and h, along with the content of each component, have been demonstrated in Additional file 6. The contents of each component and the sum of the amounts exhibited an upward trend as L_sum_ and d increased. Regarding h, the contents of the aforementioned three compounds and their cumulative total indicated a relatively weak increasing trend, while with a higher number of outliers compared to those of L_sum_ and d.

However, the content variation was only weakly explained by h. Hence, the linear regression equation was performed by L_sum_, d, and content of each component, where L_sum_ and d were used as independent variables, and perillaketone (Y_p_), egomaketone (Y_e_), isoegomaketone (Y_i_), and their sum (Y_sum_) were used as dependent variables (Additional file 7). The fitting approach employed for the content of egomaketone (Y_e_), isoegomaketone (Y_i_), and their sum (Y_sum_) demonstrated heteroscedasticity, which was addressed by applying logarithmic transformations to both the independent and dependent variables (Additional file 7). Specifically, Log10 (L_sum_) and Log10 (d) were utilized as independent variables to construct a linear regression model, while Log10 (Y_i_) and Log10 (Y_sum_) were used as dependent variables.

The adequacy of the regression equations was assessed using the regression coefficient (R^2^). The optimal linear regression equations are presented in Table 5. The findings indicated a significant association of the contents of perillaketone, isoegomaketone, egomaketone, and the total amount with both L_sum_ and d. The multivariate linear regression of L_sum_ and d showed a better fit than unary linear regression. The regression models for the total amount of these compounds (R^2^ = 0.773) and perillaketone (R^2^ = 0.698) exhibited superior performance compared to those for egomaketone (R^2^ = 0.593) and isoegomaketone (R^2^ = 0.662).

**Table 5:** The regression equations of PGTs compounds

Dependent variable	Standardised coefficients		Regression equation	R^2^	Variance test^a^
	L_sum_ [Log_10_(L_sum_)]	d [Log_10_(d)]			
Perillaketone	0.708	0.149	Y_p_ = 0.834*L_sum_ + 10.686*d + 6.971	0.698	**
Egomaketone	0.208	0.529	Log_10_(Y_e_) = 0.538*Log_10_(L_sum_) + 1.474*Log_10_(d) + 0.480	0.590	**
Isoegomaketone	0.242	0.543	Log_10_(Y_i_) = 0.667*Log_10_(L_sum_) + 1.829*Log_10_(d) + 0.814	0.662	**
Amount sums	0.511	0.405	Log_10_(Y_sum_) = 0.550*Log_10_(L_sum_) + 0.671*Log_10_(d)-1.030	0.773	**

^a^: **p < 0.01 and *p < 0.05

The standardized coefficient of the independent variable is typically used to assess its contribution to the dependent variable. The standardized coefficient of L_sum_ was larger than d in the linear regression model of perillaketone, showing that the explanation of L_sum_ was greater than d for perillaketone content. In contrast, in the linear regression model of isoegomaketone and egomaketone, the standardised coefficient of d exceeded that of L_sum_. With regards to the sum of their amounts, the standardised coefficient of d was similar to that of L_sum_, revealing that the variations in the sum of the amounts could be explained by d and L_sum_.

**Fig. 6:**
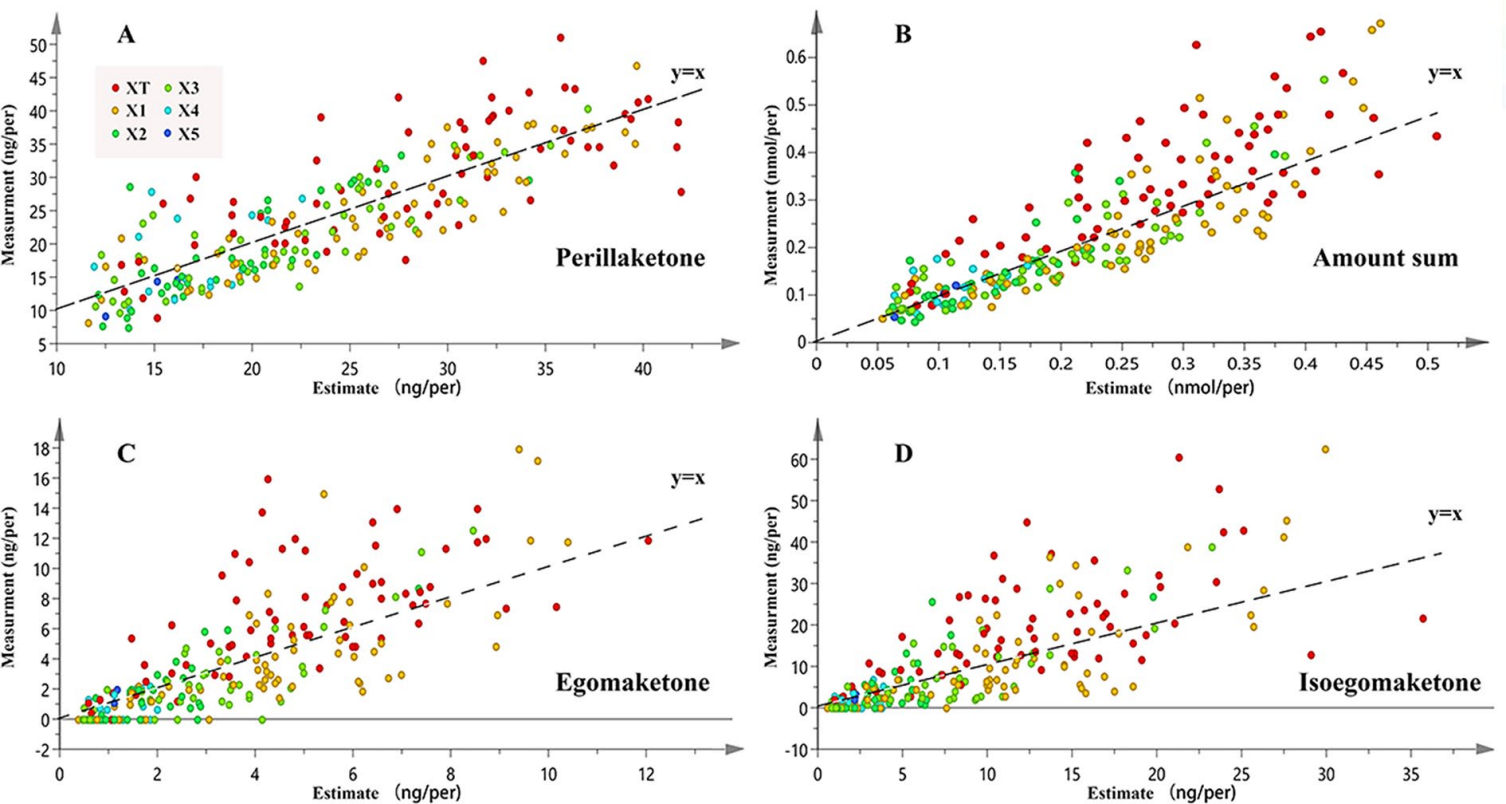
The predicted and measured contents of compounds in PGTs. XT refers to AT, BT, CT, etc.; X1 refers to A1, B1, C1, etc.; X2 refers to A2, B2, C2, etc.; X3 refers to A3, B3, C3, etc.; X4 refers to A4, B4, C4, etc.; X5 refers to A5, B5, C5, etc.

In addition, the predictive contents of perillaketone, isoegomaketone, egomaketone and the sum of their amounts were calculated based on the linear regression equation and compared with their experimental contents. As shown in Additional file 8, residuals in the linear regression equation of perillaketone were not related to specific zones, with data points of different zones were evenly distributed on both sides of the predicted contents (Fig. 6). Conversely, in the linear regression models of isoegomaketone and egomaketone, the experimental values of PGTs in zones close to the leaf margin (such as AT, BT, and CT) exceeded their predicted values, indicating the distinctive isoegomaketone and egomaketone contents in these zones. Likewise, the linear regression model of the sum of these compounds demonstrated a discrepancy between the predicted and measured contents, which was attributable to the accumulation of isoegomaketone and egomaketone.

## Discussion

### Morphology and distribution of trichomes in *P. frutescens*

In this study, three types of GTs were found in *P. frutescens*, which is consistent with previous reports [41]. The measurement results indicated that PGTs contain significantly more volatile oils than CGTs and DGTs, owing to their advantages in volume and quantity. This suggests that PGTs play a predominant role in the accumulation of volatile oils in *P. frutescens*, echoing previous research that established a proportional relationship between *P. frutescens* volatile oil content and PGT quantity [40]. Therefore, PGTs were selected as the research object for the study of the accumulation pattern of volatile oils.

It is crucial to acknowledge the fact that the metabolic products of other types of trichomes also constitute important components of other diverse plant volatile oils. Hence, it is advisable to define the research objects based on a sensible assessment of the contributions of different types of trichomes to the overall plant volatile oil in the study of other diverse plants using the proposed model. Furthermore, smaller GTs, such as DGTs and CGTs, present contrast with the surrounding background of epidermal cells, necessitating the requirement of higher spatial resolution photographs for successful automatic identification. Moreover, processing these high-resolution images requires significant computational capacity, which might limit the model's applicability. Therefore, the optimization of the model to extract the spatial phenotypes of smaller GTs without extreme computation ability will be our future study direction.

In this study, PGTs were exclusively discovered on the abaxial surface of *P. frutescens* leaves, prompting the selection of an abaxial surface as the research object for the accumulation of volatile oils. It is worth noting that the distribution of GTs on only one side of the leaf is not a universal phenomenon in plants that possess abundant glandular trichome tissue. More commonly, GTs are found on both the abaxial and adaxial surfaces of leaves [42, 44, 45]. Hence, when investigating species with GTs distributed on both sides of the leaf, the model proposed in this study requires spatial information of the GTs on both sides of the leaf, along with a cross-surface correction.

### Spatial phenotype extraction of PGTS

The number and density of GTs are crucial parameters for the quality evaluation of various crops that produce volatile oils as economic products [46]. Traditional studies have typically relied on manual counting of GTs in a random or entire area of the samples [46–48]. However, these experimental strategies often fail to ensure the effectiveness of the statistical procedure or the completeness of the information gathered. To address these limitations, we developed a workflow that utilizes the RD algorithm and a black-and-white mask. This approach facilitates the identification of PGTs from other background cells more quickly, easily, and accurately by highlighting their differences. Simultaneously, this workflow allows the extraction of more specific spatial phenotypes (such as L_0_, L_1_, and d) of PGTs by providing a coordinate representation of the spatial information of each PGT.

However, the current workflow’s manual correction and multi-step operation mode of the still impose limitations on work efficiency. Additionally, other GT phenotypes such as colour under different lighting conditions, that have not been explored as spatial information. Consequently, developing automated and encapsulated software for extracting spatial phenotypes will be a crucial research direction in the future.

### Correlation between the spatial phenotype and developmental gradient of PGTs

The development of leaves is a complex process involving cell division, differentiation, and expansion [49]. Identifying cellular developmental gradients is an important approach to understanding leaf development. The growth gradient of cells from proximal to distal, determined by the oriented division of the meristematic tissue, is widely accepted and accessible [50]. In addition, studies on *Arabidopsis* leaves have shown a gradient of gene expression laterally from the mid-vein, indicating the existence of a growth gradient from the middle to the lateral edge of the leaves [28]. Moreover, the polarity field of *Arabidopsis* leaf cells suggests that the division direction of the leafs’ epidermal cells is angled relative to the proximal–distal gradient, oriented laterally from the middle [32]. Therefore, the proximal–distal and median-lateral growth gradients form significant spatial–temporal directions for leaf development, which are consistent with the growth direction of mid-vein growth axis (MVGA) and secondary vein growth axis (SVGA) in dicotyledonous plants [34].

In this study, veins were used as spatial markers to investigate the correlation between the spatial phenotype and the developmental gradient of PGTs. The results revealed an uneven distribution of *P. frutescens* PGTs on leaves, with HDDAs concentrated at the proximal end of the MVGA and the median end of the SVGA. Here, PGTs were in the early stage of development, characterized by transparent, unfilled volatile oils. In addition, a reduction in the distribution density of PGTs was observed along the MVGA and SVGA, concomitant with the manifestation of mature characteristics, such as the presence of yellow volatile oils.

Spatial position of HDDAs aligns with the leaf meristem [50], suggesting that the leaf meristem is the generation area of PGTs and that HDDAs mark regions of meristematic activity in leaves. The leaf meristem boundary is known to be the site of cell cycle arrest, where the switch from cell proliferation to differentiation occurs [50]. The area of cell proliferation is the primary site of cell division, while cells undergo expansion and development in the differentiation zone [49, 51]. Along the MVGA and SVGA, away from the meristem, the margins and apex of leaves are positioned in the differentiation zone [52–54], and PGTs within these zones exhibit more mature characteristics, indicating their differentiation status. Furthermore, the distribution density of PGTs decreases in the differentiation zone as a result of the expansion of leaf epidermal cells.

In an investigation of PGT development in peppermint, Turner et al. [22] found that the basal zones of leaves remain meristematically active longer than the apical zones, and the cessation of PGT formation proceeds in a basipetal manner. However, these findings differ from those reported for *Montanoa. tomentosa*, where GTs are almost uniformly distributed across all zones of the leaves without any significant differences in distribution density [55].

The approach utilized in the previous research, which divided the leaf into basal, middle, and apical zones, can be conceptually regarded as segmenting the leaf into unique growth zones solely along the proximal–distal axis. Consequently, the investigation was solely concerned with the developmental gradient of GTs along the proximal–distal axis, while neglecting the gradient along the median-lateral axis. The HDDAs marking leaf meristem activity displayed a boundary that curved towards the proximal end of the leaf (Fig. 4). This finding implies that leaf zones occupying the same spatial location solely based on their proximal–distal axis may not necessarily exhibit similar developmental stages. Therefore, the successful implementation of both MVGA and SVGA in the model proposed in this study has enhanced its applicability for PGT research.

Additionally, it is noteworthy that within HDDAs, where the majority of PGTs are young, some mature PGTs can also be observed. This observation highlights the incompletely synchronized development patterns of PGTs within the same region, which aligns with the distribution of trichomes in *Arabidopsis* (Fig. 4) [21]. This asynchronous development may be attributed to the unique state of leaf primordia, where the entire leaf is considered as the meristematic region [50]. Consequently, PGTs may form patchily on the surface of the entire leaf primordia. As the epidermal tissues between PGTs proliferate, the inter-PGTs distance gradually expands. Subsequently, later-formed PGTs grow among the earlier-formed ones. Evidently, the development of PGTs involves a complex regulatory mechanism and may vary across different stages of leaf growth. Therefore, further investigation into the regulatory mechanisms governing PGTs in leaves at different growth stages is warranted.

### Correlation between the spatial phenotype and volatile oils accumulation of PGTs

Metabolic differences among GTs located in different zones of the same leaf has been observed in various plant species. In a study conducted by Voirin et al. on *Mentha* GTs, it was found that the menthone content in trichomes located at the leaf apex was higher than those at the base of the leaf [23]. Similar metabolic variation has also been observed in *Montanoa. tomentosa* [55]. However, as previously mentioned, while investigating the metabolic activity of GTs along the proximal–distal axis can provide useful insights, it is important to note that this approach may not be sufficient for accurately elucidating the developmental trajectory of GTs.

In this study, we utilized a spatial model derived from MVGA and SVGA, and found that the accumulation of perillaketone, isoegomaketone, and egomaketone in GTs was related to the spatial phenotype. Specifically, the concentration of these compounds gradually increased in the same first-level zone along the SVGA and among the different first-level zones along the MVGA. Interestingly, consistent with the findings in *Mentha *[23], there was striking variation of the volatile oil metabolism of Perilla GTs during the maturation. Perillaketone mainly accumulated in the PGTs during the early developmental stages, while the contents of isoegomaketone and egomaketone were relatively low, and in a few young PGTs, isoegomaketone and egomaketone were not undetectable (Additional file 9). As PGTs developed, the content of isoegomaketone and egomaketone gradually increased, with the proportion of volatile oil content of isoegomaketone rising faster than that of egomaketone, suggesting that isoegomaketone accumulated at a faster pace than egomaketone. Due to the rapid accumulation of egomaketone and isopletherenone, perillaketone proportion in the total volatile oil decreased, which contrasted with the trend exhibited by egomaketone and isopletherenone in the total volatile oil (Fig. 4).

**Fig. 7:**
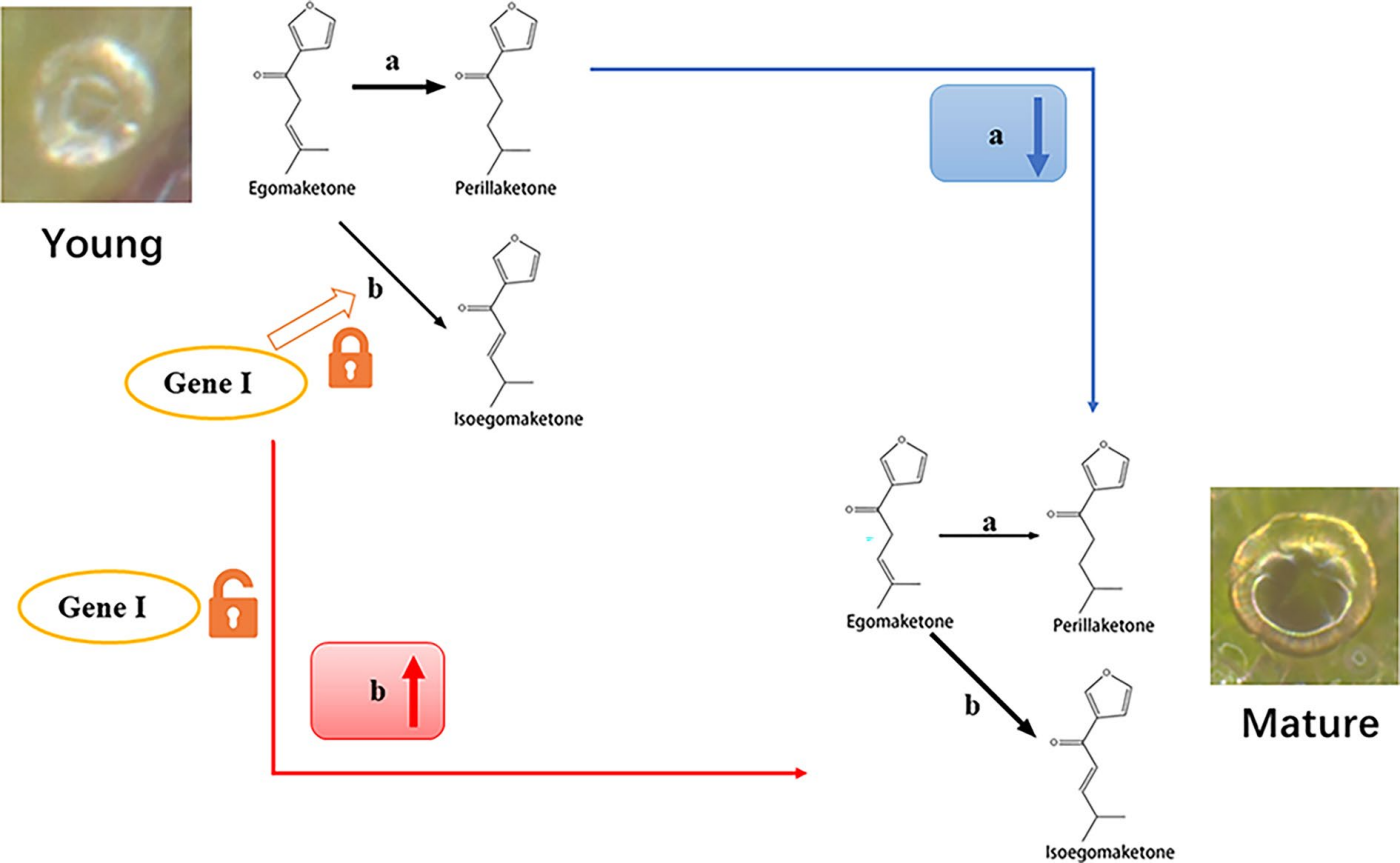
Dynamic changes of biosynthesis of perillaketone, isoegomaketone and egomaketone with the maturation of PGTs. Route **a** is the pathway from egomaketone to perillaketone; **b** is the pathway from egomaketone to isoegomaketone

Based on the results of isotope tracing experiments, egomaketone was identified as the synthesis substrate for both perillaketone and isoegomaketone [56]. Additionally, the dominant allele I (gene I) negatively regulated isoegomaketone generation from egomaketone, revealing a competitive biosynthesis between perillaketone and isoegomaketone is competitive [56]. When we combine this finding with our data on the accumulation of these three compounds, two possible initiation mechanisms can be proposed (Fig. 7). (a) The reduction in biosynthesis of perillaketone may lead to a higher utilization of egomaketone to produce isoegomaketone, while simultaneously resulting in a passive weakening of the inhibition of gene I. (b) Weakening of the inhibition of gene I beforehand may result in higher levels of egomaketone, which can generate isoegomaketone while simultaneously decreasing the rate of perillaketone synthesis. However, additional molecular biological evidence is necessary to support these hypotheses. Transcriptome and proteome analyses of GTs in different zones represents a promising avenue for future investigations. Furthermore, a previous study observed a striking variation of volatile oil metabolism, which may be attributed to the hypothesis that the production of volatile oil is regulated by light during the development and expansion of the leaf [23]. This hypothesis also provides a potential avenue for future investigations.

The results of this study also indicate that the content of β-caryophyllene in GTs varying different maturity levels demonstrated a fluctuating trend without a discernible pattern, compared to perillaketone, isoegomaketone, and egomaketone. This discrepancy can be attributed to the distinct metabolic pathways of β-caryophyllene and the aforementioned terpenes, which only intersect in the upstream stage of the metabolic pathway [4, 57, 58].

It is crucial to acknowledge some limitations in our research. Firstly, our measurement of the compounds in PGTs reflects the average situation of all PGTs within the same zone. Nevertheless, the secretion status of PGTs within the same zone was not synchronous (Fig. 4). Mass spectrometry imaging, as a method for detecting chemical components of the single-cell [59], can be explored in the future to help us further improve the accuracy of the proposed model. Moreover, our study provides a snapshot of the developmental stage of PGTs on leaves at a similar time point. Previous studies have suggested that GTs undergo a rapid maturation process, and that the developmental stage of GTs on mature leaves tends to be consistent [22–24, 42]. Therefore, we chose juvenile leaves as the research object to obtain PGTs at various developmental stages. However, it is important to recognize the necessity of exploring PGT development on a broader temporal scale. A dynamic monitoring system for PGTs development based on the proposed model could serve as a valuable tool for validating PGT development processes and could guide future research directions. However, several technical challenges still need to be addressed for developing the dynamic monitoring system, including monitoring the spatial position trajectory of PGTs at different developmental stages, quantifying and interpreting the spatial trajectory, and non-destructively monitoring secondary metabolites of PGTs.

### Correlation between the accumulation pattern of volatile oil and spatial phenotypic parameter

Previous studies on cell spatial phenotypes have identified and categorized specific cells into various zones [28, 30]. While this approach can elucidate the spatiotemporal spectrum of cell development and differentiation, it lacks a coordinated system for quantitatively describing the spatial phenotype of each cell of interest. This limitation presents a barrier to the quantitative demonstration of the conclusions obtained. In this study, the correlation between the development of PGTs and the growth axis of leaf veins provides a quantitative foundation for exploring the spatial phenotypes of PGTs and their development and metabolism.

Based on the results of PCA conducted and the findings from the linear regression models for each spatial phenotype and the content of each component, the spatial phenotype parameters L_sum_ and d were found to be able to represent the content of each component as independent variables. L_sum_ is the sum of L_1_ and L_0._ L_0_ is the distance from the germination point of each secondary leaf vein on the mid-vein to the leaf base. Thus, L_0_ indirectly represents the development stage of the entire one-level zone. And L_1_ represents the distance from the SGP to the PGT, indirectly indicating the development stage of the PGT within the one-level zone. In addition, d is the average distance between the PGT and the three adjacent PGTs. As PGTs mature, they begin to distance themselves. Therefore, the underlying logic of using L_sum_ and d to represent the development stage of PGTs is entirely different (Additional file 10).

The results from the linear regression modeling for each component (Additional file 9) indicated that while the regression models using either L_sum_ or d as independent variables, were statistically significant, exhibited lower degrees of fit compared to the multiple linear regression model that incorporated both L_sum_ and d as independent variables. Therefore, the development description model of PGTs takes the form of a combination of the L and d models.

Notably, a large regression error in the description of the composition accumulation pattern of young PGTs was observed when using the d model. This could potentially be attributed to that the generation rate of PGTs near the proximal end of the secondary leaf vein growth axis has not reached its peak, resulting in a lower PGTs density compared to adjacent high-density zones, leading to higher d values. However, the PGTs generated here are in the initial stage of growth, and the secondary metabolites have not formed a rich accumulation in those. The phenomenon of high d values coupled with low metabolite accumulation clearly contradicts the logic of the d model. Therefore, it becomes apparent that the d model is more suited to describe the growth of PGTs in their mature stage.

The residuals of isoegomaketone and egomaketone in PGTs located within the leaf margin domain exhibited values greater than zero. This implies that PGTs within the leaf margin accumulate isoegomaketone and egomaketone at a faster rate compared to those in the central domain. Alternatively, the extension rates of parameters L_sum_ and d in the leaf margin domain decreased. Within the classical description of angiosperm leaf development, the margin and central domains exhibit distinct growth and differentiation trajectories. Typically, the margin domain forms and matures earlier and is subsequently separated from the central domain due to meristematic activity. meristematic activity [49, 50, 54, 60]. Based on the underlying logic of the model, the L_sum_ and d model is more suited for tracking the growth of PGTs after the margin maturation. However, they don’t demonstrate effective tracking of the growth trajectory of the margin domain, emphasizing the importance of dynamic monitoring of the growth of PGTs for the refinement of current models, particularly during the early stages of leaf development. Additionally, the earlier maturation of the margin domain is consistent with the observed differential accumulation rates of the secondary metabolites in PGTs within the margin domains, suggesting that a correlation between PGTs development and overall leaf growth and development.

## Conclusion

In this study, we proposed a technical route that use the relative deviation values to to distinguish PGTs from the surrounding tissues and extract their spatial phenotype. Then, we employed leaf veins as the spatial reference frame to define the zones of PGTs, and constructed a regression model that links the spatial phenotypes to the growth of PGTs. The results showed that the development of PGTs is correlated with the extension and development of whole leaves, and the developmental stages of PGTs can be identified by spatial phenotypes based on the leaf veins. Moreover, we identified a striking variation in volatile oil metabolism in *P. frutescens* PGTs during the maturation. This study enhanced our comprehension of correlation between spatial phenotype and development of GTs and provides an approach for the exploration and study of the underlying regulatory mechanisms of GTs development.

### Supplementary Information

**Additional file 1: Figure S1.** T The polar field of Arabidopsis leaf epidermal cells [32] and the growth process of pinnate veins in *dicotyledons*; **A** Polarity of each epidermal cells; **B** Average polarity of epidermal cells; **C** The growth process of pinnate veins. (bar = 20 μm). The quoted pictures have been remixed.

**Additional file 2: Table S1.** The head cells diameter dimensions of various GTs in *P. frutescens.*

**Additional file 3: Figure S2.** The TIC, extracted TIC and mass spectrogram of GC-MS analysis of PGTs. **A** The TIC of standards; **B** The TIC of extraction; **C** The TIC of extract ions 95.1; **D** The TIC of extract ions 132.8; **E** The TIC of extract ions 71.1; **F** The MS2 of perillaketone; **G** egomaketone; **H** isoegomaketone; **I** β-caryophyllene.

**Additional file 4: Figure S3.** The PCA analysis of spatial phenotypes of PGTs from different zones. **A** Perillaketone; **B** Isoegomaketone; **C** Egomaketone; **D** β*-*caryophyllene.

**Additional file 5: Figure S4.** The PCA analysis of the spatial phenotypes of PGTs from different zones. Different color represented the amount sum.

**Additional file 6: Figure S5.** The relationship between spatial phenotypes (L_sum_, d and h) and the contents of the main compounds.

**Additional file 7: Table S2.** Linear regression model equations.

**Additional file 8: Figure S6.** Distribution of residues of perillaketone, isoegomaketone, and egomaketone in different zones.

**Additional file 9: Table S3.** Spital phenotypic parameters and compound contents of each zone.

**Additional file 10: Figure S7. **Schematic diagram of growth state characterization models of PGTs (L model, d model).

## Data Availability

The data supporting the findings of this study are available from the corresponding author (Qinan Wu) upon request.

## Abbreviations

GTs: Glandular trichomes
PGTs: Peltate glandular trichomes
GC–MS: Gas chromatography mass spectrometry
RD: Relative deviation value
PGP: Primary growth point
SGP: Secondary growth point
LTP: Leaf top point
PCA: Principal component analysis
CGTs: Capitate glandular trichomes
DGTs: Digitiform glandular trichomes
HDDAs: High-density distribution areas
MVGA: Mid-vein growth axis
SVGA: Secondary veins growth axis
