# Supplementary figures and images for: Applying quantitative spatial phenotypes analysis to the investigation of peltate glandular trichomes development pattern in Perilla frutescens
Source: Plant Methods. 2023 Aug 25;19:88. doi: 10.1186/s13007-023-01072-4 (PMC10464211; doi:10.1186/s13007-023-01072-4)

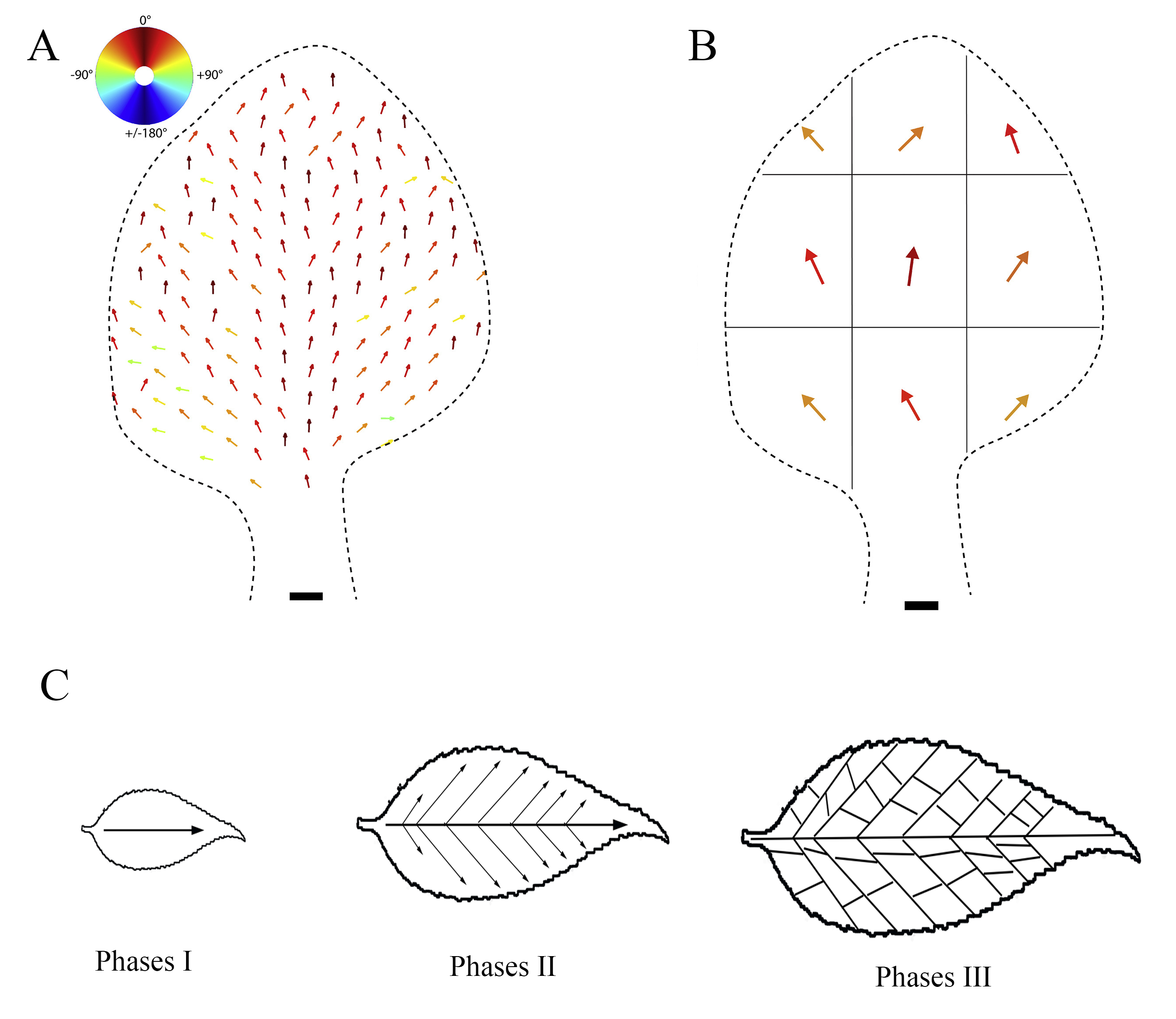

Supplement: Supplementary file 1 — Additional file 1: Figure S1. T The polar field of Arabidopsis leaf epidermal cells [32] and the growth process of pinnate veins in dicotyledons; A Polarity of each epidermal cells; B Average polarity of epidermal cells; C The growth process of pinnate veins. (bar = 20 μm). The quoted pictures have been remixed. [file 13007_2023_1072_MOESM1_ESM.tif]

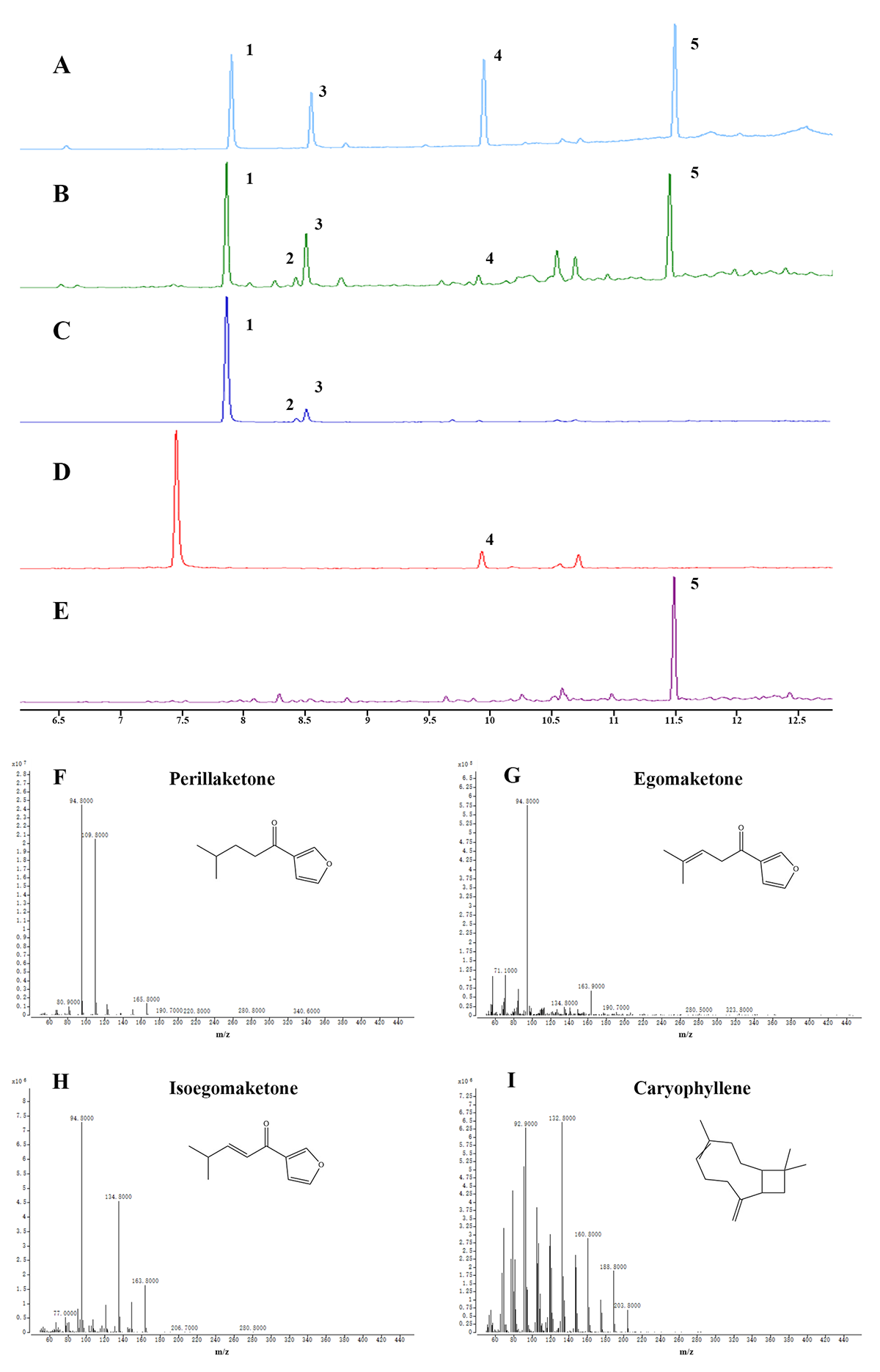

Supplement: Supplementary file 3 — Additional file 3: Figure S2. The TIC, extracted TIC and mass spectrogram of GC-MS analysis of PGTs. A The TIC of standards; B The TIC of extraction; C The TIC of extract ions 95.1; D The TIC of extract ions 132.8; E The TIC of extract ions 71.1; F The MS2 of perillaketone; G egomaketone; H isoegomaketone; I β-caryophyllene. [file 13007_2023_1072_MOESM3_ESM.tif]

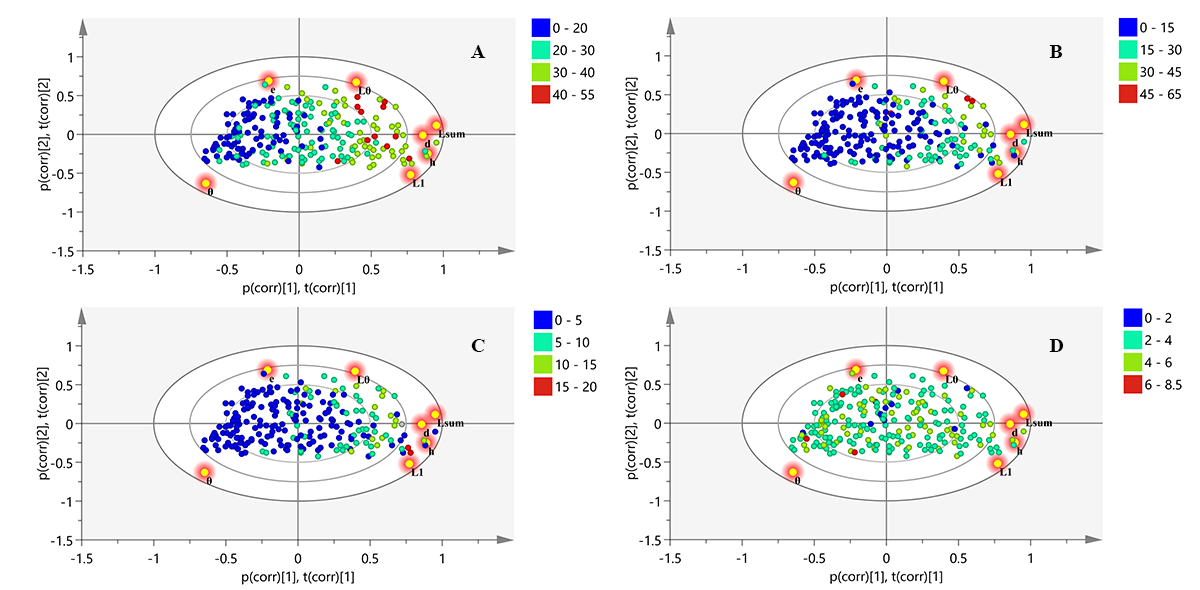

Supplement: Supplementary file 4 — Additional file 4: Figure S3. The PCA analysis of spatial phenotypes of PGTs from different zones. A Perillaketone; B Isoegomaketone; C Egomaketone; D β-caryophyllene. [file 13007_2023_1072_MOESM4_ESM.tif]

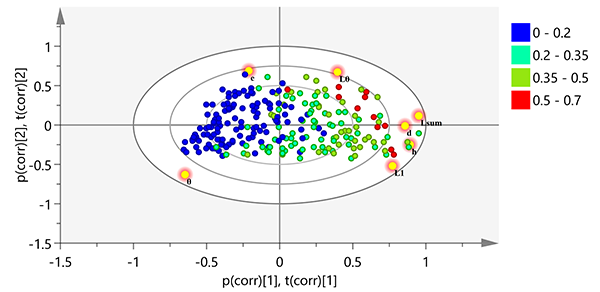

Supplement: Supplementary file 5 — Additional file 5: Figure S4. The PCA analysis of the spatial phenotypes of PGTs from different zones. Different color represented the amount sum. [file 13007_2023_1072_MOESM5_ESM.tif]

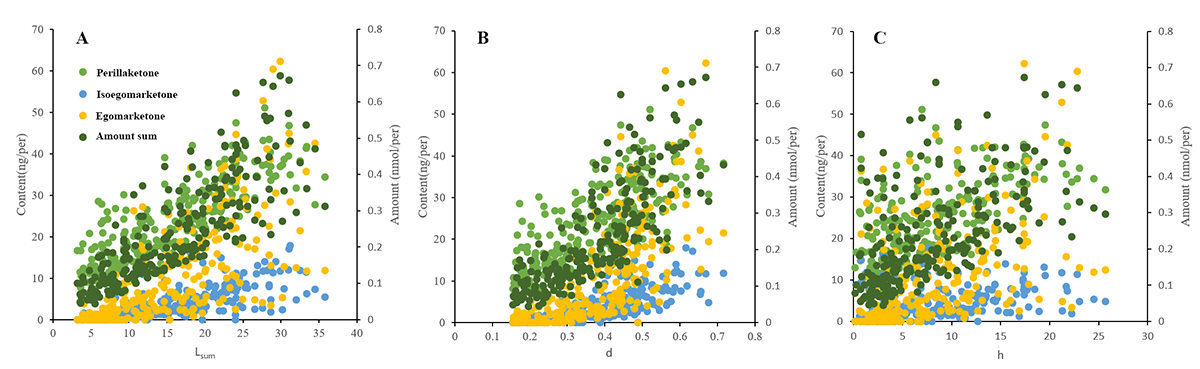

Supplement: Supplementary file 6 — Additional file 6: Figure S5. The relationship between spatial phenotypes (Lsum, d and h) and the contents of the main compounds. [file 13007_2023_1072_MOESM6_ESM.tif]

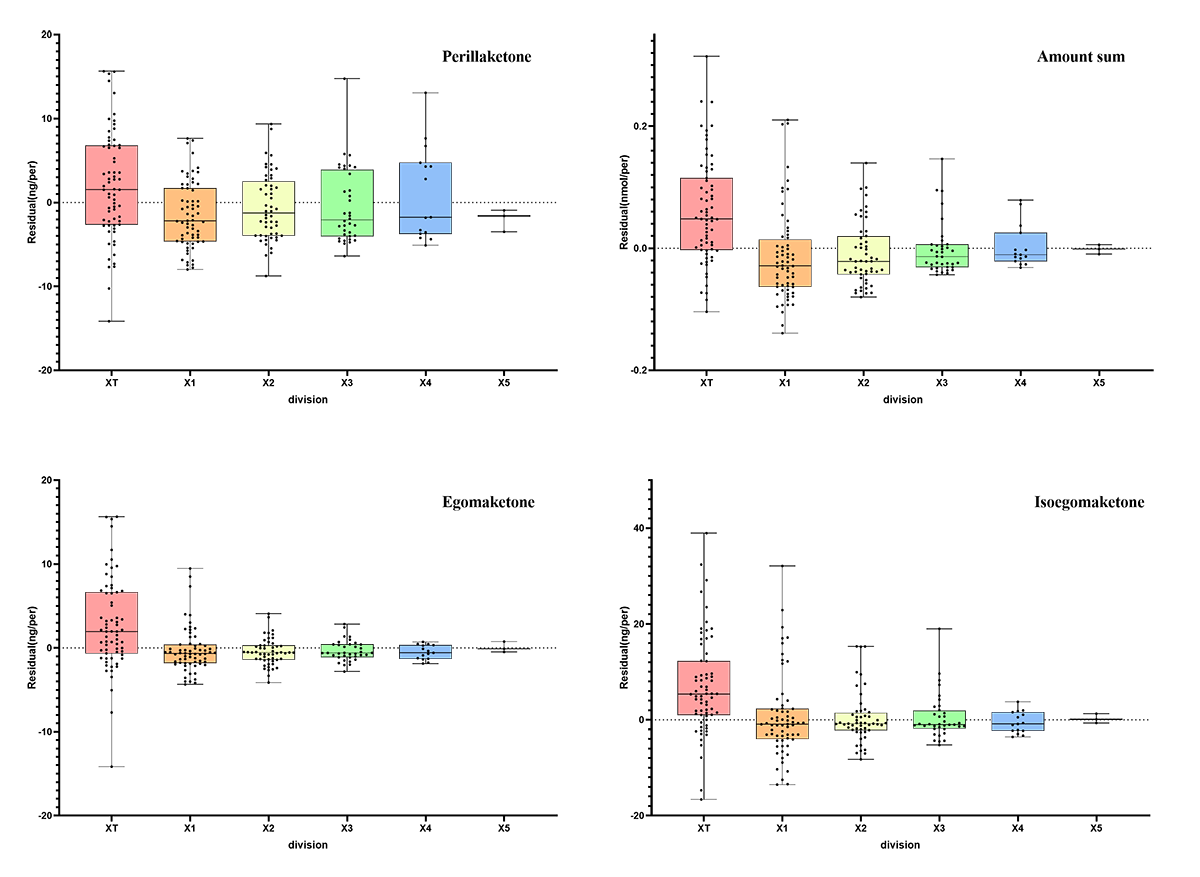

Supplement: Supplementary file 8 — Additional file 8: Figure S6. Distribution of residues of perillaketone, isoegomaketone, and egomaketone in different zones. [file 13007_2023_1072_MOESM8_ESM.tif]

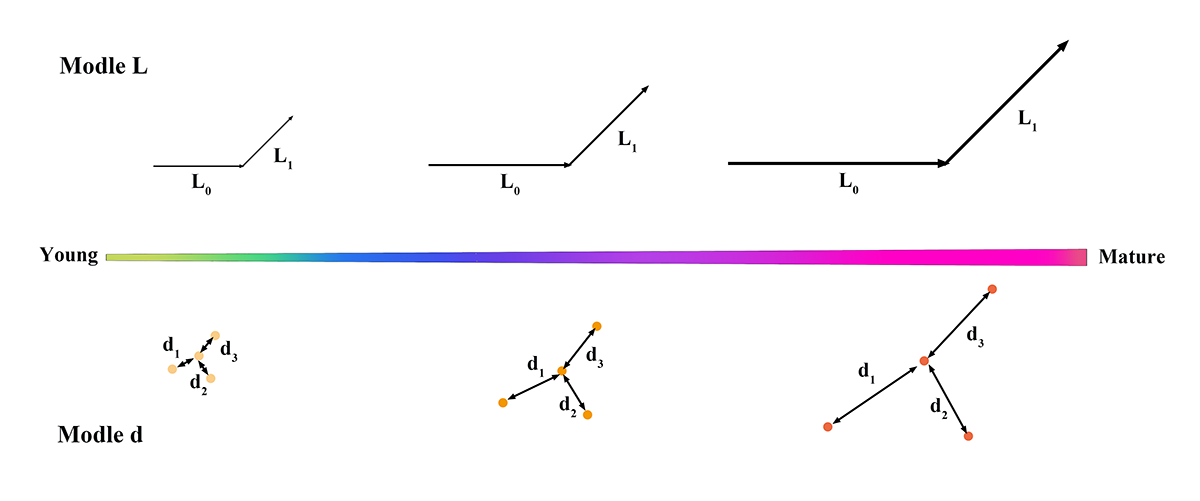

Supplement: Supplementary file 10 — Additional file 10: Figure S7. Schematic diagram of growth state characterization models of PGTs (L model, d model). [file 13007_2023_1072_MOESM10_ESM.tif]
